# Differential Expression of Epstein–Barr Virus Sequences in Various Breast Cancer Subtypes

**DOI:** 10.3390/genes16070756

**Published:** 2025-06-27

**Authors:** Alexander Blanchard, Namarig Elmalih, Perpetua Muganda

**Affiliations:** 1 Applied Science and Technology Ph.D. Program, North Carolina A&T State University, Greensboro, NC 27411, USA; apblanch@aggies.ncat.edu; 2 Department of Biology, North Carolina A&T State University, Greensboro, NC 27411, USA; nelmalih@aggies.ncat.edu

**Keywords:** Epstein–Barr virus, human gammaherpesvirus 4, breast cancer, viral oncogenesis, triple-negative breast cancer, HER2+ breast cancer, viral miRNA, computational genomics, gene expression analysis, bioinformatics, RNA-seq

## Abstract

**Background/Objectives**: Breast cancer (BC) is the most common source of new cancer diagnoses among women and the second leading cause of cancer-related deaths in this group. The role of viral factors in the etiology, heterogeneity, and pathogenesis of this disease and its subtypes has not been incontrovertibly determined. Thus, in this study we began to address this problem by testing the hypothesis that the oncogenic Epstein–Barr virus (EBV) plays a role in this process. The approach involved determining the differential expression and predicted role of EBV gene sequences present in various subtypes of breast tumors as compared to those in control normal tissues. **Methods**: We utilized existing deep sequencing RNA-seq datasets derived from seventeen breast tumors and three control normal breast tissue samples to investigate the differential expression of EBV gene sequences. **Results**: We report three-fold higher levels of normalized total EBV-expressed sequences in tumors as compared to in control breast tissue. We also demonstrate differential expression of EBV gene transcript sequences in four categories of 26 known genes in breast cancer tumors as compared to that in normal breast tissue controls. Tumor-specific expression of EBV gene transcript sequences localized to seventeen genes; of these, tumor-specific EBV gene transcript-expressed sequences localizing to nine genes were strongly differentially expressed in a breast cancer subtype-specific manner. Furthermore, in a proof-of-concept investigation, we report, for the first time, that functional analysis of the differentially expressed integrated EBV transcript sequences demonstrate the capacity of these sequences to generate novel EBV miRNAs. We conclude that these integrated EBV sequences could potentially play a role in the pathogenesis of BC and its most aggressive subtypes. The functional role of these findings is currently under study.

## 1. Introduction

Breast cancer (BC) is the most common type of cancer among women and the second leading cause of cancer related deaths within this group [1,2]. This complex disease exists in four distinct heterogeneous molecular subtypes that are associated with different clinical outcomes and responses to treatment [3,4,5,6,7,8]. The etiology and molecular basis of the heterogeneity and pathogenesis of breast cancer and its most aggressive subtypes are not completely understood [7,9].

The four main intrinsic heterogeneous molecular subtypes of breast cancer include luminal A, luminal B, human epidermal growth factor 2 positive (HER2)-enriched, and triple-negative breast cancer (TNBC)/basal-like [5,6,7]; of these, the human epidermal growth factor 2 positive (HER2+) and the triple-negative/basal breast cancer (TNBC/basal) subtypes are the most aggressive [9]. HER2-positive breast cancers represent 10–15% of all breast cancers, and are estrogen receptor-negative, progesterone receptor-negative, and HER2 receptor-positive. They have a poor prognosis with a fast pace of growth compared to luminal breast cancer [5]. HER2 breast cancer tumor heterogeneity has been associated with poor prognosis, tumor relapse, and anti-HER2 targeted therapy resistance [10]. The TNBC/basal subtype, which accounts for 10–15% of the breast cancer cases, is the most heterogeneous group of breast cancer tumors. These tumors are estrogen receptor-negative, progesterone receptor-negative, and HER2-negative, with high proliferation rates [11,12,13,14]. They are the most clinically aggressive breast cancer tumors, with poor survival rates; they also lack targeted therapy, tend to be resistant to chemotherapy, and display a high risk of relapse [5]. The TNBC/basal breast cancer subtype is subcategorized into seven different subtypes; these include basal-like 1 (BL1), basal-like 2 (BL2), mesenchymal-like (M), mesenchymal stem-like (MSL), immunomodulatory (IM), luminal androgen receptor (LAR), and claudin-low type (CL) [5,14,15]. Current evidence has implicated the influence of hereditary susceptibility, epigenetics, the action of noncoding RNA, tumor heterogeneity, and tumor microenvironment on the aggressiveness of breast cancer subtypes [11,12,16,17,18,19,20,21]. The inter-tumor and intra-tumor heterogeneity of BC contributes to post-treatment tumor relapse, and is controlled by multiple factors [22,23,24]. It is highly likely that oncogenic viruses, such as Epstein–Barr virus (EBV, human herpesvirus 4), play a role in the etiology, heterogeneity, and molecular pathogenesis of the aggressive breast cancer subtypes.

Epstein–Barr virus (EBV, human gammaherpesvirus 4) is a highly ubiquitous dsDNA containing a virus that is estimated to infect 95% of the human population [25,26]. This group I carcinogen possesses an etiological role in some forms of lymphomas and epithelial cancers, such as nasopharyngeal carcinoma and gastric carcinoma [26,27]. In this role, both latent and lytic replication cycles of EBV are involved in oncogenesis [28,29], and EBV functions through various molecular mechanisms [26,30]. This virus can activate cellular oncogenes and inactivate tumor suppressor genes, disrupt cell cycle regulation, increase proliferation, and decrease apoptosis. EBV can trigger pro-inflammatory responses and oxidative stress, evade and suppress the immune system, and promote immune evasion within the tumors; this further drives the carcinogenic process [28,29,31,32]. Furthermore, EBV can alter the extracellular matrix, promoting tumor cell growth and invasion. In addition, EBV also increases angiogenesis, thus enhancing tumor migration and invasion. The EBV genome sequences can integrate into the human genome, inducing genomic instability, mutations, and chromosome aberrations [26,33]; in fact, the EBV genome can disappear from tumor cells when it is no longer needed, leaving only short integrated sequences [34]. Furthermore, EBV can contribute to the cancer process by modifying the epigenome through methylation and histone modifications [35]. EBV-encoded miRNAs can also modulate cell growth, apoptosis, and the immune response through their target genes [36]. Finally, EBV can alter the tumor microenvironment and enhance cancer progression through the transfer of encoded proteins and miRNAs in the form of exosomes [37]. Although the EBV oncogenic mechanisms in epithelial malignancies are complex, and have not been fully elucidated [26], these mechanisms point to the capacity of this virus to mediate oncogenesis in similar epithelial malignancies, such as breast cancer. In fact, the presence of EBV or its products in breast cancer tumors has been associated with increased aggressiveness, epithelial–mesenchymal transition, worse prognosis, increased stemness and tumor heterogeneity, inflammation, immune modulation, and resistance to chemotherapy [38,39,40,41,42]; all of these conditions are found in aggressive tumors.

MicroRNAs (miRNAs) are small non-coding RNAs (18–24 nucleotides long) involved in regulating gene expression at the post-transcriptional level [43]. Similar to cellular miRNAs, EBV miRNAs have been implicated in the pathogenesis of EBV-associated cancers; within these tumors, EBV miRNAs positively modulate tumorigenesis, invasion, metastasis, and drug resistance, as well as inflammation and immune evasion [44,45,46,47,48,49,50,51]. For example, EBV miRNA expression was found to be sufficient for NPC tumorigenesis [52]. Expression of a single EBV miRNA was found to sufficiently inhibit p53 expression to maintain latency and promote tumorigenesis in both NPC and GC tumors [53]. Some EBV miRNAs expressed in NPC were found at levels comparable to those of host miRNAs, and sequence variants known as isomiRs were found in some EBV miRNAs [54,55]. Dysregulation of host pathways by EBV miRNAs in NPC resulted in the altered expression of host miRNAs and the further reduction in immune function [45]. Although EBV-encoded miRNAs play an important role in NPC and GC, there have been no previous published reports on the existence and role of any EBV-encoded miRNAs in breast pathogenesis. Therefore, there is a critical need to understand the existence and role of EBV-encoded miRNAs in breast cancer.

Studies focused on determining the prevalence and role of EBV sequences in breast cancer have utilized traditional molecular biology techniques (such as PCR and in situ hybridization), as well as deep sequencing approaches to quantitate EBV gene expression and EBV DNA [56,57,58,59,60,61,62,63,64,65,66,67,68,69]. A 2022 meta-analysis of EBV–breast tumor association studies identified 19 studies reporting a positive association between EBV and breast tumors, and 9 studies reporting no EBV expression in breast tumors [56]. Most of the studies on EBV in breast cancer have delivered variable and contradictory results, due to the reliance of these studies on polymerase chain reaction (PCR) and in situ hybridization techniques [58,62,65]. The variable nature of existing reports is also attributed to the existence of specific EBV strains in certain populations and geographical locations [25,70,71,72]. Studies that have utilized the RNA-seq approach also reported little to no significant expression of EBV sequences in breast cancer cases; under these experimental conditions some investigators found EBV transcripts in gastric carcinoma tumors or EBV DNA in breast cancer [58,59,60,64]. All these observations suggest that EBV expression in breast cancers is very low compared to the results for gastric carcinomas. In fact, no complete EBV genome has yet been found or sequenced in breast cancer cases, although this has been accomplished for other EBV-associated gastric and nasopharyngeal carcinomas [57,61,63,66,67,68,69]. In addition, the EBV genome can disappear from breast cancer tumors [40,73,74], leaving only remnants of short EBV sequences integrated into the host genome [75,76]. These short-integrated sequences will be hard to detect, either at the DNA level or as transcribed sequences; this may then account for at least some of the variable reporting that accompanies EBV detection by various methods, including short-read deep sequencing approaches. The EBV sequences within the breast cancer tumors are also variable due to tumor heterogeneity and to the fact that EBV is likely to display different stages of latency, integration and is lost once the tumor has been initiated [34]. The high involvement of ncRNAs (such as the fact that miRNAs are not being studied or that they are harder to detect), low maintenance of viral infection after tumorigenesis, and high tumor heterogeneity are likely significant contributing factors to the ongoing controversy regarding EBV-driven oncogenesis in breast cancer. Although EBV has been epidemiologically linked to breast cancer, the role of this virus in the etiology, heterogeneity, and pathogenesis of breast cancer is not completely understood, and there is a critical need to solve this problem [7,9,26,38,77,78].

In this study, we worked towards determining the role of EBV in the etiology, heterogeneity, and pathogenesis of breast cancer and its most aggressive subtypes. We utilized RNA-seq and associated techniques since they represent the best approach for our studies; this is due to the ability of these techniques to determine the identity, structure, prevalence, and quantitation of genome-wide expressed sequences and transcripts [65]. We investigated the differential expression and predicted the function of EBV gene sequences present in various subtypes of breast tumors as compared to that in control normal tissues; an existing deep sequencing RNA-seq dataset derived from 17 breast tumors and 3 control normal breast tissue samples was utilized. Our findings demonstrate higher levels of normalized total EBV-expressed sequences in tumors as compared to control breast tissue. We also demonstrate differential expression of EBV gene transcript sequences in four categories of 26 known genes in breast cancer tumors as compared to normal breast tissue controls. Tumor specific expression of EBV gene transcript sequences localized to seventeen genes; of these, tumor specific EBV gene transcript expressed sequences localizing to nine genes were strongly differentially expressed in a breast cancer subtype specific manner. Furthermore, in a proof of concept investigation, we report for the first time that functional analysis of the differentially expressed integrated EBV transcript sequences demonstrate the capacity of these sequences to generate novel EBV miRNAs.

## 2. Materials and Methods

### 2.1. RNA-Seq Datasets Utilized

RNA-Seq deep sequencing data (PRJNA227137) [79] utilized for this study was obtained from the NCBI/SRA database This dataset contains 17 breast tumors (6 TNBC, 6 Non-TNBC, 5 HER2+) and 3 control breast tissues. Ribo-depleted whole transcriptome RNA was prepared from all samples using the RiboMinus Eukaryote Kit (Invitrogen, Carlsbad, CA, USA). RNA libraries were prepared and sequenced using standard Illumina protocols.

### 2.2. RNA-Seq Data Analysis

Analysis of RNA-seq data was conducted utilizing the Galaxy Server [80]. Raw fastq data were quality controlled by utilizing the FastQC 0.74 tool, and sequencing adapters were removed using the Trim Galore! 0.6.10 [81] tool in automatic detection mode. After further FastQC quality control, cleaned sequencing reads were aligned to the Epstein–Barr virus (EBV) reference genome (NC_007605.1) by utilizing the HISAT2.2.1 tool with default settings. Alignment BAM files were assembled into transcripts by utilizing StringTie 2.2.3 in guided assembly mode with the EBV genome as the reference. The Output Gene Abundance Estimation File Advanced Option was used to produce tabular files containing expression data in FPKM values for all annotated genes; all other options were analyzed at default values. Genes with expressed sequences appearing in at least two tumors of a given subtype were selected for differential expression analysis. Differential expression of the EBV gene sequences were visualized by utilizing the Morpheus heatmap tool. Expressed gene sequences were sorted into four groups: expressed in all samples, but upregulated in tumors, uniquely expressed in all tumors, differentially expressed between specific tumor subtypes, and downregulated in tumors compared to controls. GraphPad Prism version 11 was used to compare FPKM expression levels and the prevalence (percentage of non-zero reads) of expressed EBV gene sequences in the various experimental and control groups. The expression and identity of the EBV gene sequences was confirmed by visualizing the successful alignment of these sequences against the EBV genome utilizing The Integrative Genomics Viewer (IGV) 2.1.1.2 [82].

### 2.3. Identification of EBV Gene Expressed Sequence Hotspots

Expression hotspots (as visualized through IGV) were identified as locations at which multiple tumors aligned to the EBV genome with the same expressed transcript sequence. Alternatively, expression hotspots were sequences which were uniquely expressed in a single breast cancer subtype. Hotspot sequences were quantitated and further analyzed for sequence variations between normal control tissues and breast cancer subtypes.

### 2.4. Identification of Novel EBV miRNAs Potentially Generated from the EBV Gene-Expressed Sequence Hotspot Regions

EBV genome-aligned BAM files obtained from control and breast tumor miRNA-seq data (PRJNA482141, NCBI SRA) were visualized against the EBV genome using IGV. IGV visualized EBV miRNAs differentially expressed between control and breast tumors and localizing within the EBV gene-expressed sequence hotspot regions were noted for each gene. Two control and two tumor samples selected for further analysis for each hotspot sequence were presented as IGV visualizations, and the relationship of the selected EBV IGV miRNAs to the hotspot coordinates within IGV were shown. One differentially expressed miRNA localizing within each EBV-expressed sequence hotspot region was depicted on the IGV visualization by a star; the identified IGV miRNAs were then analyzed to determine their origination from the EBV gene-expressed sequence hotspot regions in a series of steps, as follows. First, the reference sequence of the forward strand of each miRNA marked with a star and shown in IGV was recorded; this was labeled as Refseq for each miRNA. Next, the actual variant sequence of the same miRNA was determined and contrasted with the reference sequence (Refseq) by bolding and underlining the variant and reference residues; the actual miRNA containing the variant information was named IGV miRNA, since it is shorter than the typical 21–22 nucleotide miRNA sequence. Since all the hotspots and corresponding miRNAs being studied were located on the EBV genome reverse strand, each IGV miRNA sequence was converted to its reverse complement and named IGV miRNA_RC. The EBV aligned BAM files containing each IGV miRNA_RC were converted to FASTA format utilizing the Galaxy Samtools fastx tool (Galaxy version 1.15.1 + galaxy2) [80]. The most abundant complete (20–22 nucleotides long) miRNA FASTA sequence containing each IGV miRNA_RC was then retrieved from the corresponding EBV aligned BAM to FASTA-converted miRNA sequences; the Filter FASTA tool (Galaxy Version 2.3) or the Grep tool (Galaxy Version 9.3 + Galaxy 1) were utilized for this purpose, and the obtained full length miRNA for each hotspot was named miRNA_RC. Each complete length miRNA_RC for each hotspot was utilized to retrieve potential miRNA precursor sequences from the corresponding hotspot RNA-seq EBV-aligned BAM files after they had been converted to a FASTA sequence. The miRNA_RC sequence and the hotspot sequence were mapped to the retrieved corresponding 60 bp miRNA precursor; the 60 bp sequence was confirmed as the miRNA_RC precursor if it contained the complete miRNA sequence. The 60 bp RNA-seq hotspot containing sequence was further confirmed as the miRNA-RC precursor if it generated the full length miRNA-RC after processing through the miRNA prediction tool MatureBayes [83], which converts miRNA precursors to their corresponding mature 20–22 nucleotide-long miRNAs. The obtained MatureBayes-predicted miRNAs were compared against the identified miRNA-RC to confirm the generation of the novel EBV miRNAs from the corresponding EBV-expressed sequence hotspot. This workflow is summarized in Supplementary Figure S5.

### 2.5. Statistical Analysis

Data are shown as means. A one-sided Student’s *t*-test was performed to compare means using GraphPad Prism version 10.0. *p* < 0.05 was used to determine statistical significance.

## 3. Results

### 3.1. Expression of Total EBV Sequences Was Higher in Breast Cancer Tumors as Compared to the Results for Control Normal Breast Tissues

**Figure 1:**
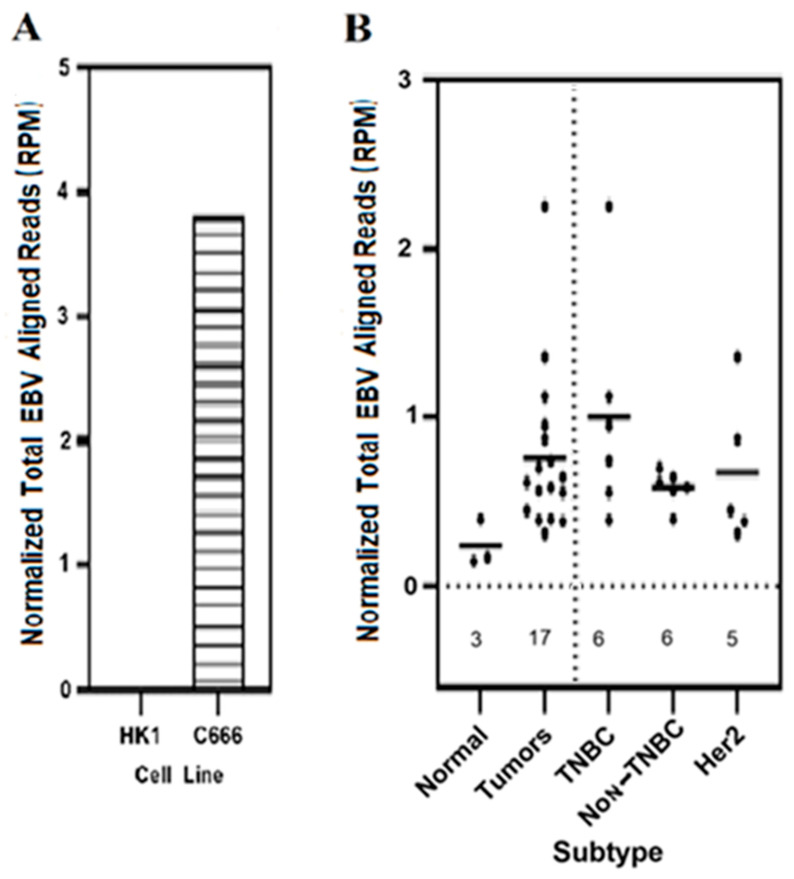
Quantitation of total EBV-expressed sequences in normal controls and in various breast cancer tumor subtypes. RNA-seq datasets obtained from the NCBI SRA Bioproject PRJNA227137 conducted by Eswaran et al. [79] were trimmed and aligned against the EBV genome in fasta format by utilizing the HISAT2 aligner, as described in Methods. The normalized total EBV-aligned expressed sequences in reads per million (RPM) quantitated from the BAM files for each sample are shown. (**A**) EBV-negative HK1 and EBV-positive C666 cell lines (SRR 7757113 and SRR 7757114, respectively, taken from pre-existing NCBI SRA RNA-seq data) used as negative and positive controls for the presence of EBV-expressed sequences, respectively. (**B**) Control normal breast tissues, all breast tumors, and the various breast cancer subtypes within all breast tumors utilized are shown. The number of the samples analyzed for each category are shown above the x-axis. TNBC, triple negative breast cancer subtype; Non-TNBC, luminal A and B; HER2; human epidermal growth factor receptor 2 positive breast cancer subtype.

We determined the prevalence of total EBV-expressed sequences in the different subtypes of breast cancer tumors as compared to that in control normal breast tissues (Figure 1). Normalized total EBV-expressed sequence reads were readily detected in the EBV-positive C666 tumor cell line, but were undetectable in the EBV-negative control normal cell line HK1 (panel A). Under these alignment conditions, normalized total EBV-expressed sequences were detected in 100% of the control and breast cancer tumor samples (panel B); the expression levels, however, were at least three-fold higher in the tumors as compared to those in control normal tissue. Overall, these results demonstrate the preferential expression of normalized total EBV-expressed sequences in breast cancer tumors over control normal tissues. A slight but not statistically significant preference for TNBC breast tumors was observed.

### 3.2. Expression of EBV Gene Transcript Sequences Were Elevated in Various Breast Cancer Tumor Subtypes as Compared to That in Control Normal Breast Tissues

**Figure 2:**
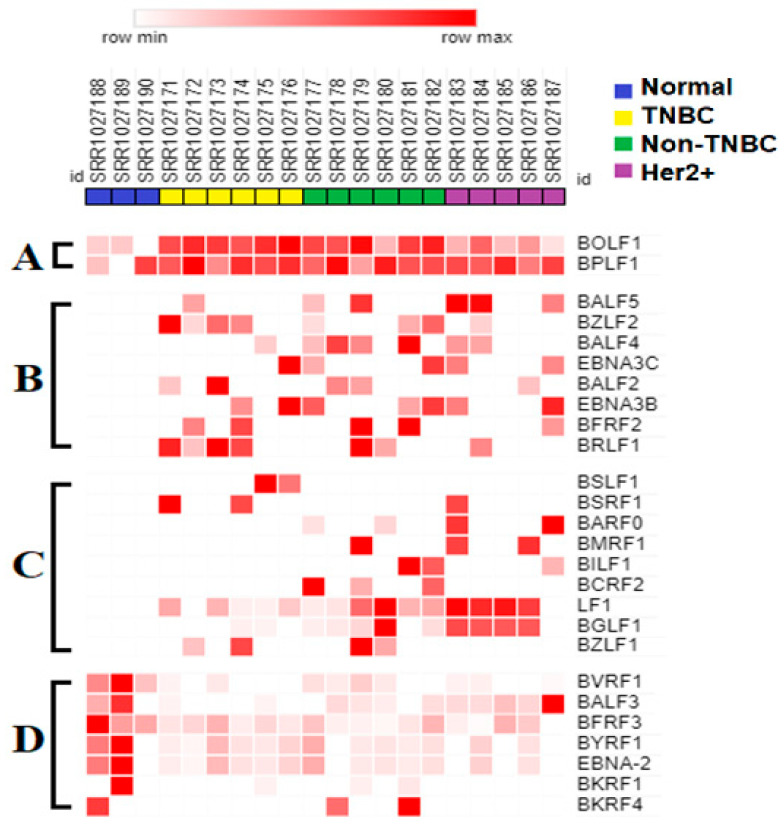
Expression of EBV gene transcript sequences in normal controls and in various breast cancer tumor subtypes. Trimmed raw RNA-seq reads from normal and various breast cancer subtypes (as shown in Figure 1B) were aligned against the EBV genome in FASTA format using OK. BAM file reads were assembled into transcripts and quantified by utilizing the StringTie tool and the EBV genome in gene feature format. A heat map depicting the relative quantity (in FPKM values) of the specific EBV gene transcript-expressed sequences is shown, and is stratified as follows: (**A**) EBV gene transcript sequences expressed in normal controls and tumors; (**B**) EBV gene transcript sequences expressed in all tumors but undetectable in normal control breast tissue; (**C**) EBV gene transcript sequences differentially expressed in the various breast cancer subtypes, but undetectable in normal controls; (**D**) EBV gene transcript sequences downregulated in the breast cancer tumor subtypes compared to in normal controls.

The differential expression of EBV gene transcript sequences in the various breast cancer tumors as compared to normal breast tissue was determined (Supplementary Figure S2, Figure 2). A total of 67 known EBV gene transcript sequences were expressed, but no expression was detected from 28 known EBV genes (Supplementary Figure S2). Twenty-six of the EBV gene transcript sequences expressed in at least two tumors of any given subtype or controls are shown in Figure 2. These expressed EBV gene transcript sequences were subdivided into four groups (panels A–D), according to their patterns of expression. Two EBV gene transcript sequences were expressed in all samples but were upregulated in breast cancer tumors as compared to normal control breast tissues (panel A). Eight EBV gene transcript sequences were expressed specifically in breast cancer tumors, but not in normal control breast tissues (panel B). Nine EBV gene transcript sequences were either uniquely expressed or strongly upregulated in specific breast cancer tumor subtypes (panel C). In addition, seven EBV gene transcript sequences were downregulated in breast cancer tumor subtypes compared to in normal breast tissue controls (panel D). Overall, these results demonstrate the differential expression of specific EBV gene transcript sequences in various breast cancer tumor subtypes.

### 3.3. Seven EBV Gene Transcript Sequences Were Downregulated in Breast Tumors Compared to in Control Normal Breast Tissue

The differential expression of gene transcript sequences expressed in normal control breast tissue at greater average levels than in breast tumors identified seven downregulated EBV gene sequences (Figure 2D, Supplementary Figure S3). BFRF3 was expressed at the highest levels for these sequences, with 100% expression in controls and expression in 94% of breast tumors at approximately 1/3 the level. EBNA-2 and BYRF1 sequences, which overlap significantly due to a largely shared reading frame, were both expressed in 67% of controls and 65% and 76% of tumors, respectively; levels of expression in the tumors averaged around 25% of the levels detected in the normal control breast tissues. BKRF4 sequences were expressed at comparable levels between the control and breast tumor samples, but in only 12% of tumors compared to in 33% of controls. These results also demonstrate the differential expression and downregulation of specific EBV gene transcript sequences in breast tumors compared to in normal control breast tissues.

### 3.4. BOLF1 and BPLF1 EBV Gene Transcript Sequences Expressed in All Samples Are Upregulated in Breast Cancer Tumors Compared to in Normal Control Breast Tissue

**Figure 3:**
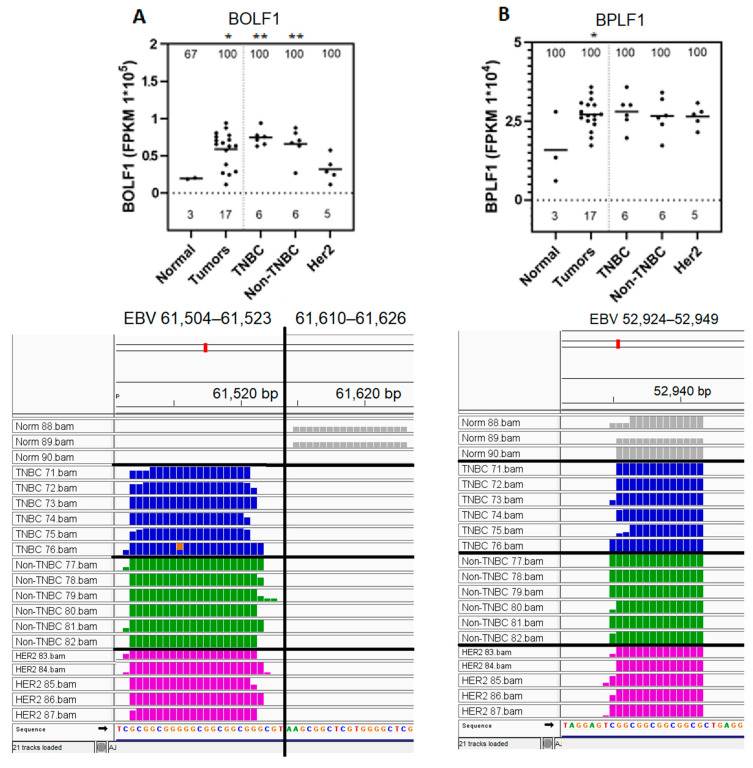
Upregulated expression of EBV BOLF1 and BPLF1 gene transcript sequences in breast cancer tumors as compared to in control normal samples. BOLF1 and BPLF1 EBV gene transcript sequences quantitated in Figure 2A for each sample are shown in graphs (top panels). The number of the samples analyzed for the graphical representation of each subtype is shown above the x-axis. The % of the positive samples expressing each EBV gene transcript sequence for each sample subtype is shown at the top of each the graph. The analysis of the corresponding read coverage contained in the bam files is shown through IGV representation (bottom panels). (**A**). BOLF1 gene sequences expression in graphical (top panel) and IGV visualization of the corresponding bam files (bottom panel). (**B**). BPLF1 gene sequences expression in graphical (top panel) and IGV visualization of the corresponding bam file (bottom panel). Statistical testing was performed for each subtype against controls; subtypes marked with * are upregulated with *p* < 0.05 and ** with *p* < 0.01.

The differential expression and nature of the BOLF1 and BPLF1 EBV gene transcript sequences found to be expressed in normal and tumor samples were further analyzed (Figure 3). This was accomplished by further analyzing the results obtained as described in Figure 2, panel A, through graphical representation (Figure 3, top panel), and IGV visualization of the corresponding bam files (Figure 3, bottom panel). BOLF1 transcript-expressed sequences were prevalent in 100% of the tumors and subtypes, as compared to in 67% of the normal control breast tissue; levels of BOLF1 transcript sequence expression were approximately three-fold higher in the TNBC and non-TNBC breast cancer tumors as compared to in control normal tissue (panel A, top panel). IGV visualization demonstrated that BOLF1-expressed sequences in all tumors clustered into 61,504–61,523 map units of the EBV genome. Conversely, BOLF1 transcript sequences expressed in normal breast tissues clustered into the 61,610–61,626 map units of the EBV genome (panel A, bottom panel). BPLF1 transcript sequences were prevalent in 100% of all control normal breast tissue and all tumors, with expression levels approximately 1.5-fold higher in tumors compared to control samples (panel A, top panel). BPLF1-expressed sequences localized within the 52,931–52,943 region of the EBV genome (Figure 3B, bottom panel). Collectively, these results demonstrate the upregulation of BOLF1 and BPLF1 transcript sequences in all breast tumors compared to control normal tissues. These results also demonstrate that the expression of BOLF1 transcript sequences maps to different regions of the EBV genome in breast cancer tumors compares to control normal breast tissues.

### 3.5. Eight EBV Gene Transcript Sequences Were Expressed in Breast Tumors and Not in Normal Breast Tissue Samples

**Figure 4:**
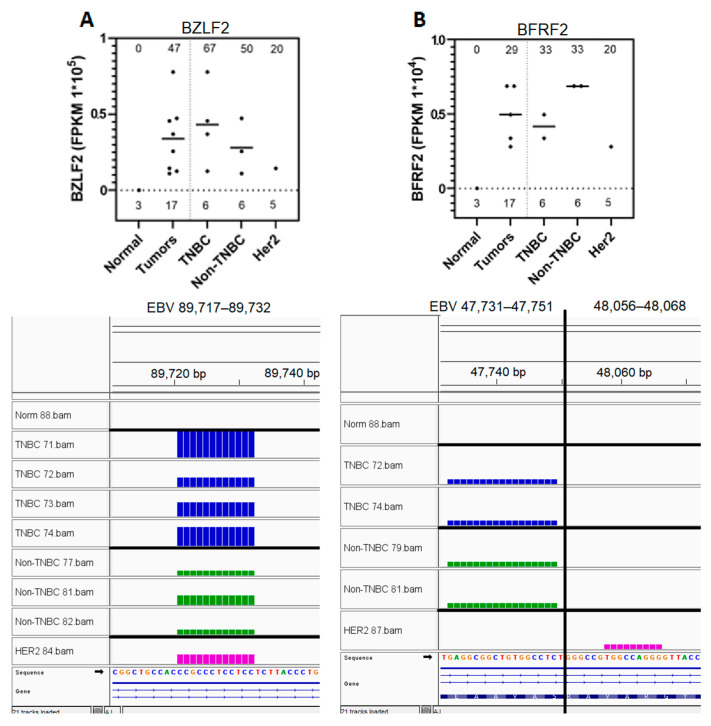
Expression of EBV BZLF2 and BFRF2 gene sequences in breast tumors and subtypes and not in normal breast tissue controls. Expression analysis of the EBV gene sequences in normal controls and in various breast cancer tumor subtypes was determined as explained under Figure 2B. (**A**) BZLF2 gene sequence expression in graphical format (**top panel**) and IGV visualization of the corresponding BAM file read coverage (**bottom panel**). (**B**) BFRF2 gene sequence expression in graphical format (**top panel**) and IGV visualization of the corresponding BAM file read coverage (**bottom panel**). The number of samples analyzed for the graphical representation for each subtype is shown above the x-axis. The % of the positive samples expressing each EBV gene sequence for each subtype is shown at the top of each graph.

Eight EBV gene transcript sequences were expressed in breast tumors, with no detectable expression observed in normal breast tissues (Figure 2B, Supplementary Figure S4); of these transcript sequences, BZLF2 sequences were expressed in 47% of the tumors, while BALF4, EBNA3B, and BRLF1 were expressed in 41% of all tumors. BZLF2 and BFRF2, uniquely expressed in 47% and 29% of tumors, respectively, were selected for further analysis (Figure 4). BZLF2 is one of the most prevalent tumor-specific sequences. BZLF2 and BFRF2 transcript sequences are both expressed in hotspots, while transcript sequences of many other genes, such as BRLF1, are expressed across different locations. BZLF2 transcript sequences were expressed in 67% of TNBCs, 50% of non-TNBCs, and 20% of HER2 breast cancer subtypes. All BZLF2 transcript-expressed sequences localized to the 89,717–89,732 region of the EBV genome (Figure 4A).

BFRF2 gene transcript sequences were expressed in 33% of the TNBC and non-TNBC samples, as well as in 20% of the HER2 samples. All BFRF2 expressed sequences localized to two nearby regions (47,731–47,751 and 48,056–48,068) within the EBV reference genome. The high prevalence and expression of the BZLF2 and BFRF2 gene transcript sequences in tumors point to a potential importance of these expressed sequences in tumorigenesis; this is also supported by the observation that each gene-expressed sequence exists as a hotspot localizing to a common region of the EBV genome in all breast tumors.

### 3.6. EBV BGLF1 and LF1 Gene Transcript Sequences Are Preferentially Expressed in HER2-Positive Tumors and Not in Normal Breast Tissue Controls

**Figure 5:**
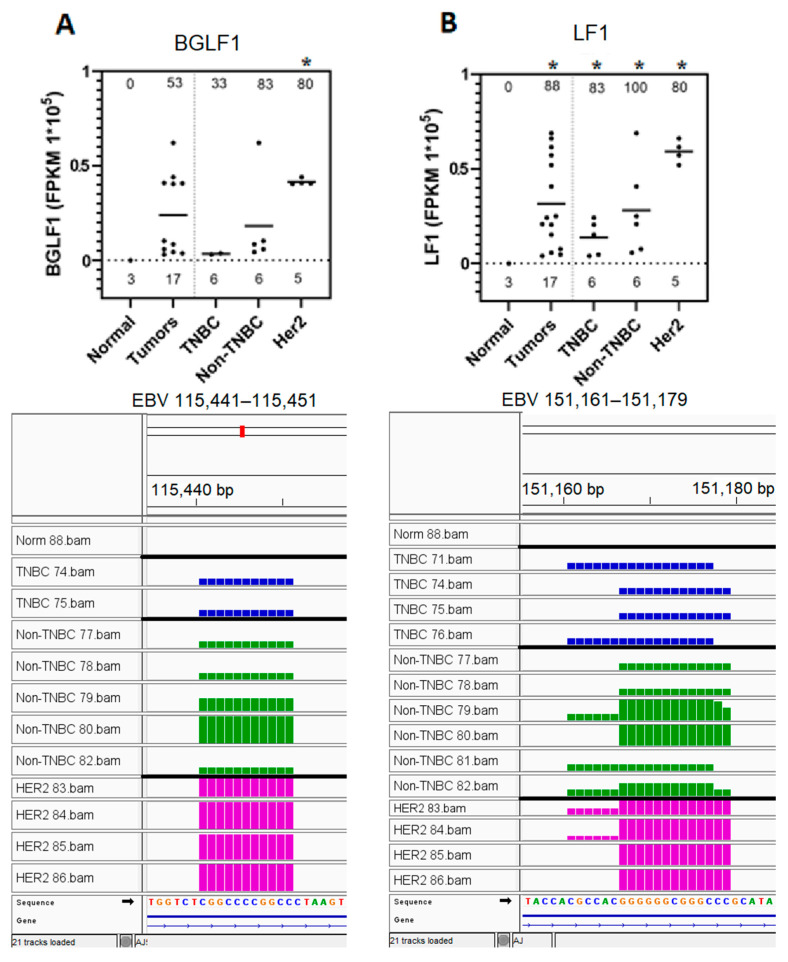
Preferential expression of EBV BGLF1 and LF1 gene sequences in HER2-positive tumors and not in normal breast tissue controls. Expression analysis of the EBV gene sequences in normal controls and in various breast cancer tumor subtypes was determined as explained under Figure 2C. (**A**) BGLF1 gene sequence expression in graphical format (**top panel**) and IGV visualization of the corresponding BAM file read coverage (**bottom panel**). (**B**) LF1 gene sequence expression in graphical format (**top panel**) and IGV visualization of the corresponding BAM file read coverage (**bottom panel**). The number of samples analyzed for the graphical representation for each subtype is shown above the x-axis. The % of the positive samples expressing each EBV gene sequence for each subtype is shown at the top of each graph. Statistical testing was performed for each subtype against controls; subtypes marked with * are upregulated with *p* < 0.05.

The differential expression of genes either uniquely expressed or upregulated in specific breast cancer subtypes was investigated (Figure 2C, Supplementary Figure S4). EBV BGLF1 and LFI gene sequences were found to possess the highest expression levels, and were thus selected for further analysis. The results obtained as described in Figure 2, panel C were analyzed through graphical representation (top panel) and IGV visualization of the corresponding BAM file (bottom panel), as shown in Figure 5. Both BGLF1 and LF1 sequences were expressed in 80% of Her2+ tumors, and were upregulated approximately two-fold higher in the Her2+ breast cancer tumor subtypes as compared to the results for all other tumors (Figure 5, panels A and B, respectively); under these experimental conditions, no expression was observed in normal breast tissue controls. Both gene transcript sequences were expressed at the lowest levels in the TNBC subtype, while the non-TNBC subtype contained intermediate expression levels (top panels A and B). IGV visualization and further analysis revealed that the BGLF1-expressed sequence was aligned to the 115,441–115,451 map units of the EBV genome. The LF1-expressed sequences primarily aligned within the 151,161–151,179, region of the EBV genome, although a variant of the sequence expressed primarily in Her2+ tumors is 6nt shorter (151,166–151,179) (bottom panels A and B). These results suggest that the LF1 and BGLF1 hotspot sequences may play a role in the differentiation of the HER2+ subtype within breast tumors.

### 3.7. EBV BSRF1 Gene Transcript Sequences Were Preferentially Expressed in Triple-Negative Breast Cancer and HER2 Subtypes

**Figure 6:**
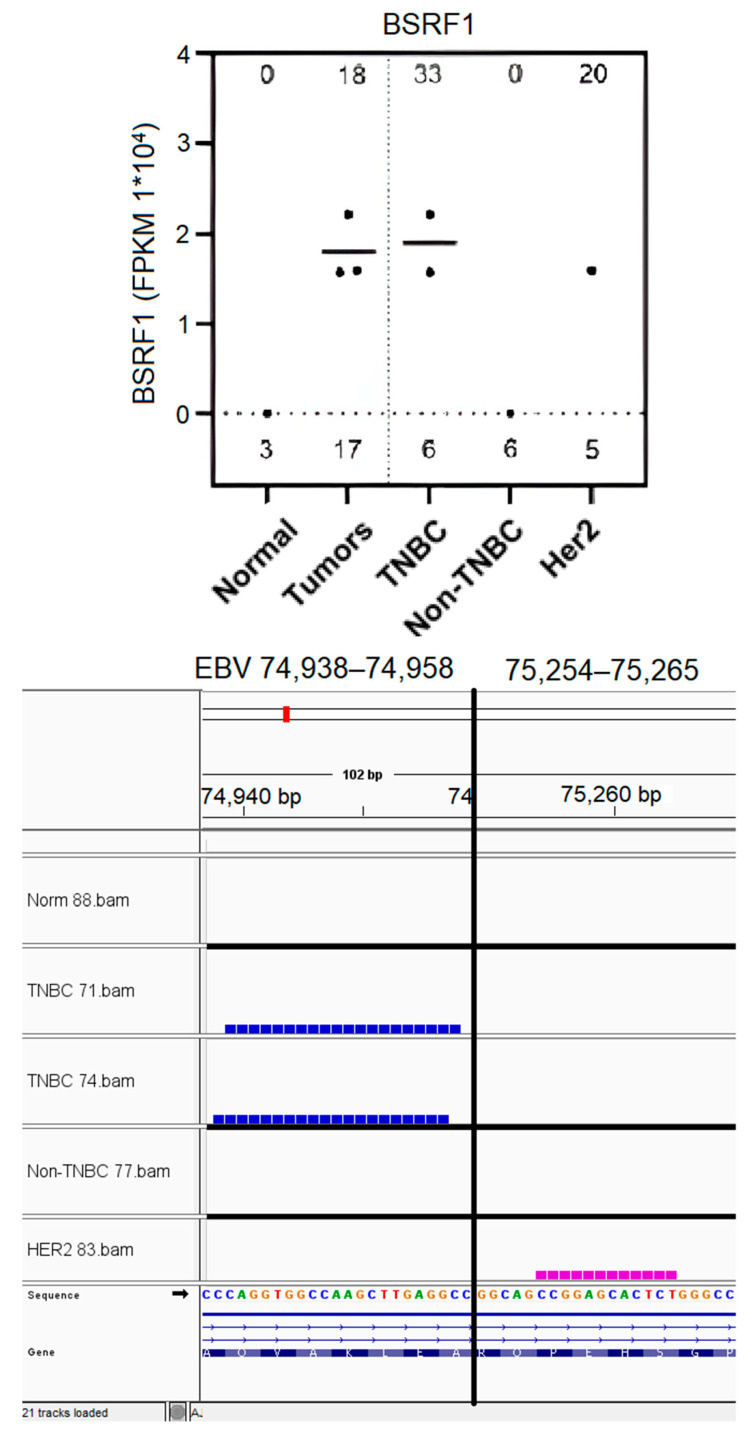
Preferential expression of EBV gene BSRF1 transcript sequences in TNBC and HER2 breast tumors. EBV BSRF1 gene transcript sequences quantitated (as in Figure 2C) for each sample are graphically represented (**top panel**). The coverage contained in the corresponding BAM file is shown through IGV visualization (**bottom panel**). The number of the samples analyzed for the graphical representation for each subtype is shown above the x-axis. The % of the positive samples expressing each EBV gene sequence for each subtype is shown at the top of each graph.

BSRF1 gene transcript sequences were expressed in 33% of TNBC tumors and 20% of HER2+ tumors; these sequences were not detected in non-TNBC tumors or control normal breast tissues. The BSRF1 transcript sequence expression levels were not significantly different between the TNBC and HER2 breast cancer subtypes (Figure 6, top panel). The BSRF1 transcript-expressed sequences localized to the EBV genome in the TNBC tumors around map units 74,938–74,958 (Figure 6, bottom panel). Conversely, the BSRF1-expressed sequences localized to the EBV genome in the HER2 tumor around EBV map units 75,254–75,265. Collectively, these results demonstrate the expression of different specific BSRF1 gene transcript sequences in the TNBC subtypes as compared to in the HER2+ breast cancer tumor subtype.

### 3.8. EBV BSLF1 Gene Transcript Sequences Are Expressed Specifically in Triple-Negative Breast Cancer Subtypes

**Figure 7:**
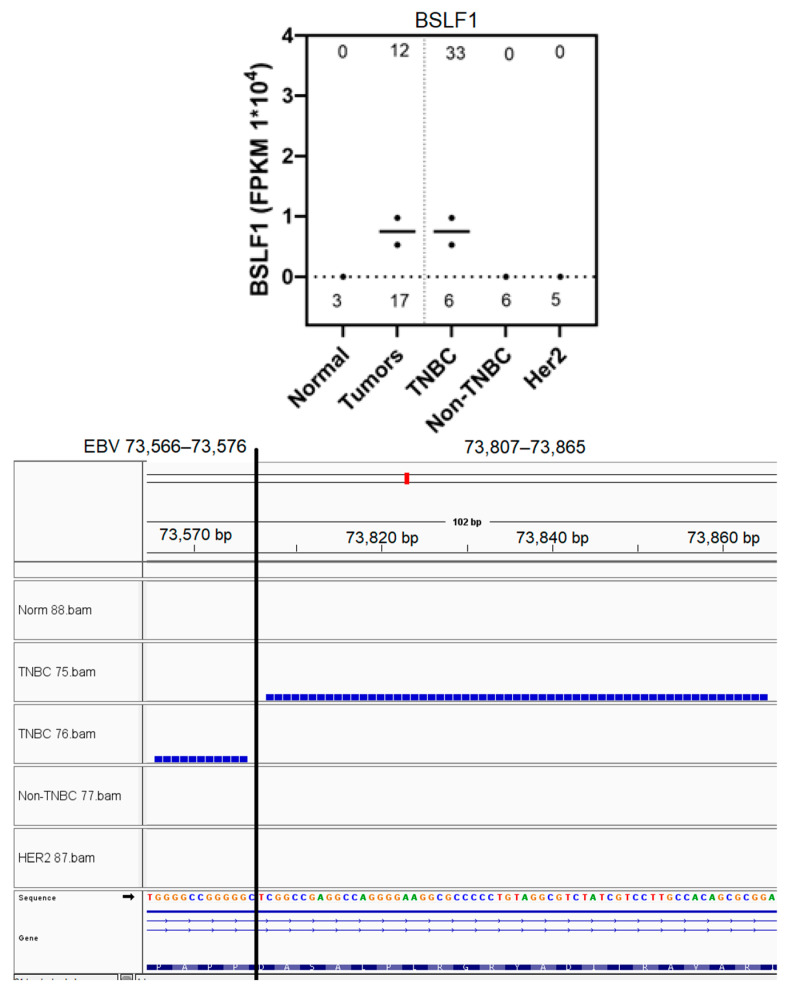
Specific expression of EBV BSLF1 gene transcript sequences in TNBC tumors. EBV BSRF1 gene sequences quantitated (as in Figure 2C) for each sample are graphically represented (**top panel**). The coverage contained in the corresponding BAM file is shown through IGV visualization (**bottom panel**). The number of the samples analyzed for the graphical representation for each subtype is shown above the x-axis. The % of the positive samples expressing each EBV gene sequence for each subtype is shown at the top of each graph.

BSLF1 gene transcript sequences were specifically expressed in 33% of TNBC tumors, but these expressed sequences were not detected in other breast cancer subtypes or control tissues (Figure 7, top panel). The BSLF1 transcript-expressed sequences detected localized within the EBV genome at map units 73,566–73,576 in one TNBC tumor, and at map units 73,807–75,865 in the second TNBC tumor. Since TNBC subtypes are heterogeneous and exist in six different molecular subtypes [84], these results suggest TNBC subtype-specific expression of the BSLF1 gene transcript sequences.

### 3.9. Functional Analysis of EBV Transcript Sequences Detects Putative Novel miRNAs

**Figure 8:**
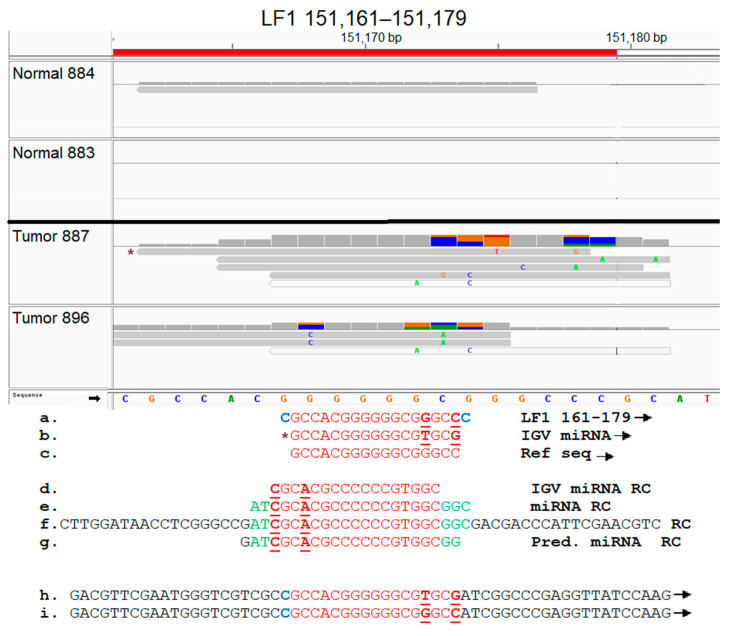
Identification of a novel EBV miRNA potentially generated from the LF1 (151,161–151,179) hotspot sequences. Breast tumor miRNA-seq sequences were aligned against the EBV genome, as described in Methods. The aligned BAM files were visualized using IGV, and the EBV IGV miRNAs localizing within the LF1 hotspot regions are shown (**top panel**). For each sample utilized, bam file coverage is shown on the top line, and the EBV IGV miRNA reads are shown in the lower section. The sequence of one IGV miRNA marked by * was analyzed further. (**a**). LF1 complete hotspot sequence. (**b**). EBV IGV miRNA sequence. (**c**). Reference EBV IGV miRNA sequence. (**d**). EBV IGV miRNA in reverse complement (RC) due to alignment on reverse strand (**e**). Complete EBV IGV miRNA sequence retrieved from EBV-aligned miRNA BAM file. (**f**). RNA-seq sequence containing the complete 22 nucleotide miRNA sequence shown in (**e**). (**g**). miRNA predicted by processing sequence shown in (**f**) through MatureBayes software. (**h**,**i**) Forward strand of the reverse complement shown in (**f**), with and without SNPs, respectively. Note: Nucleotide sequences different between reference and actual miRNA sequence are bolded and underlined. RC and forward arrow denotes reverse complement and forward strand, respectively. Red sequences are found in hotspot and miRNA. Blue indicates the sequence appears in hotspot but not in miRNA. Green and black sequences indicate putative human genes, with green sequences found in miRNA but not in hotspot.

**Figure 9:**
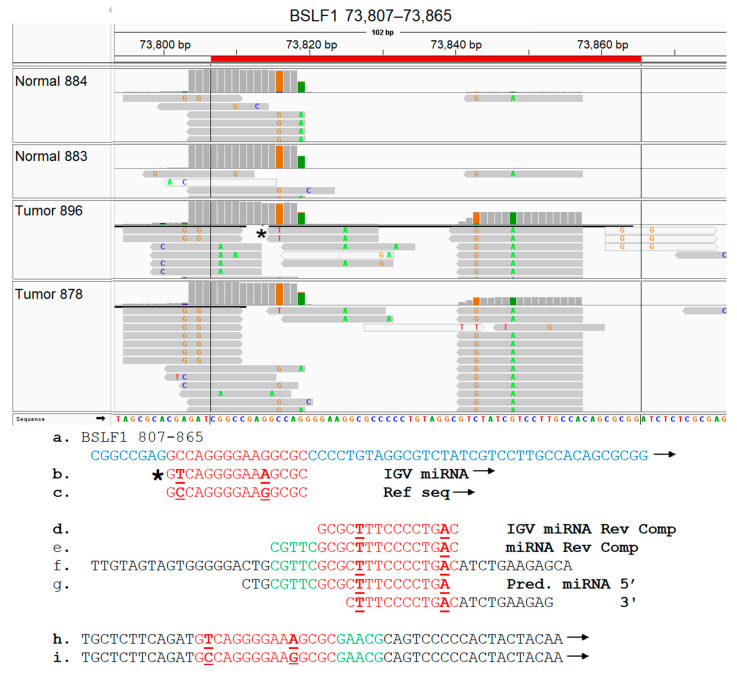
Identification of a novel EBV miRNA potentially generated from the BSLF1 (73,815–73,829) hotspot sequences. Breast tumor miRNA-seq sequences were aligned against the EBV genome, as described in Methods. The aligned BAM files were visualized using IGV, and the EBV IGV miRNAs localizing within the BSLF1 hotspot regions are shown (**top panel**). For each sample utilized, bam file coverage is shown on the top line, and the EBV IGV miRNA reads are shown in the lower section. The sequence of one IGV miRNA marked by * was analyzed further. (**a**). BSLF1 complete hotspot sequence. (**b**). EBV IGV miRNA sequence. (**c**). Reference EBV IGV miRNA sequence. (**d**). EBV IGV miRNA in reverse complement (RC) due to alignment on reverse strand (**e**). Complete EBV IGV miRNA sequence retrieved from EBV-aligned miRNA BAM file. (**f**). RNA-seq sequence containing the complete 22 nucleotide miRNA sequence shown in (**e**). (**g**). miRNA predicted by processing sequence shown in (**f**) through MatureBayes software. (**h**,**i**) Forward strand of the reverse complement shown in (**f**), with and without SNPs, respectively. Note: Nucleotide sequences different between reference and actual miRNA sequence are bolded and underlined. RC and forward arrow denotes reverse complement and forward strand, respectively. Red sequences are in hotspot and miRNA. Blue indicates sequence appears in hotspot but not in miRNA. Green and black sequences are putative human sequences with green sequences found in miRNA but not in hotspot.

**Figure 10:**
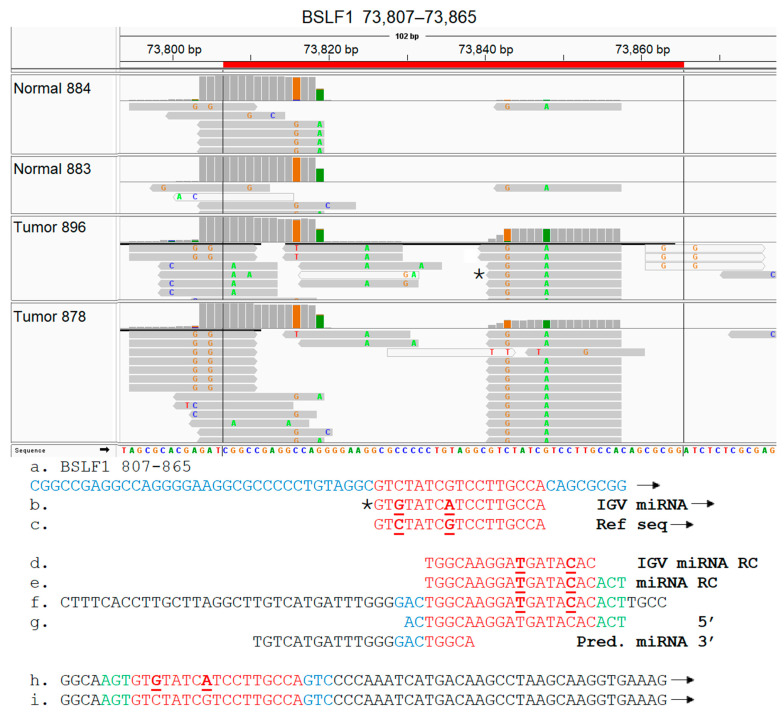
Identification of a novel EBV miRNA potentially generated from the BSLF1 (73,841–73,857) hotspot sequences. Breast tumor miRNA-seq sequences were aligned against the EBV genome, as described in Methods. The aligned BAM files were visualized using IGV, and the EBV IGV miRNAs localizing within the BSLF1 hotspot regions are shown (**top panel**). For each sample utilized, bam file coverage is shown on the top line, and the EBV IGV miRNA reads are shown in the lower section. The sequence of one IGV miRNA marked by * was analyzed further. (**a**). BSLF1 complete hotspot sequence. (**b**). EBV IGV miRNA sequence. (**c**). Reference EBV IGV miRNA sequence. (**d**). EBV IGV miRNA in reverse complement (RC) due to alignment on reverse strand (**e**). Complete EBV IGV miRNA sequence retrieved from EBV-aligned miRNA BAM file. (**f**). RNA-seq sequence containing the complete 20 nucleotide miRNA sequence shown in (**e**). (**g**). miRNA predicted by processing sequence shown in (**f**) through MatureBayes software. (**h**,**i**) Forward strand of the reverse complement shown in (**f**), with and without SNPs, respectively. Note: Nucleotide sequences different between reference and actual miRNA sequence are bolded and underlined. RC and forward arrow denotes reverse complement and forward strand, respectively. Red sequences are in hotspot and miRNA. Blue indicates sequence appears in hotspot but not in miRNA. Green and black sequences are putative human sequences, with green sequences found in miRNA but not in hotspot.

Select EBV gene-expressed sequence hotspots regions were subjected to functional analysis, based on tumor specificity and expression levels. To accomplish this, the hypothesis that the short EBV gene transcript-expressed sequences generate novel miRNAs was tested. The experimental approach was conducted as described in Methods, and as summarized in the flow diagram shown in Supplementary Figure S5; due to experimental constraints, only two tumor samples were tested in this proof-of concept investigation. After identifying the alignment of miRNA sequences to the hotspot, complete sequences of these IGV miRNAs were retrieved from EBV-aligned miRNA BAM files for three select hotspots; all three miRNAs contained non-virus sequences, implying integration of viral sequences to the host genome. The miRNAs aligned to the LF1 (151,161–151,179) hotspot were identified in both tumors and one control sample shown, but the number of reads detected was lower in the control sample (Figure 8). The LF1 miRNA present in the breast tumors contained two single nucleotide polymorphisms (SNPs) which were absent in the control sample. miRNAs were aligned to the BSLF1 (73,807–73,865) hotspot at multiple locations. A tumor-specific miRNA aligned to the BSLF1 hotspot at 73,815–73,829; this miRNA was confirmed by the MatureBayes prediction (Figure 9). The third miRNA aligned to the BSLF1 hotspot at 73,841–73,857 and was also confirmed through MatureBayes prediction. Expression levels of this miRNA were dramatically higher in the tumors as compared to in the controls, and expression appears to be elevated in tumors compared to in the controls (Figure 10). Taken together, these proof-of-concept results clearly demonstrate that the integrated differentially expressed EBV hotspot sequences found in breast tumors can generate novel EBV miRNA.

## 4. Discussion

The role of the Epstein–Barr virus (EBV) in breast cancer (BC) etiology, heterogeneity, and pathogenesis has not been incontrovertibly determined. Thus, in this study, we began to address this problem by determining the differential expression, nature, and potential role of EBV gene sequences present in various subtypes of breast tumors as compared to that in control normal tissues. We identified a three-fold higher level of normalized total EBV-expressed sequences in breast tumors as compared to in control breast tissues. Differential expression of EBV transcript sequences in breast cancer tumors as compared to in normal breast tissue controls was observed within four categories of 26 known genes. Two EBV gene (BOLF1, BPLF1) transcript sequences were expressed in all samples but were upregulated in breast cancer tumors as compared to in normal control breast tissues; in contrast, seven EBV gene transcript sequences were downregulated. Eight EBV gene transcript sequences were expressed specifically in breast cancer tumors, but not in normal control breast tissues. Nine EBV gene transcript sequences were strongly upregulated or not expressed in specific breast cancer tumor subtypes; no expression was observed in control breast tissues for these sequences. LF1 and BGLF1 EBV gene transcript sequences were expressed in 88% and 53% of the BC tumors, respectively; both gene transcript sequences were preferentially expressed in 80% of Her2+ BC tumors at approximately two-fold higher levels when compared to the average values of all tumors. BC subtype-specific expression of different versions of BSRF1 transcript sequences was observed in 20% of HER2+ and 33% of the triple-negative BC tumors. BSLF1 gene transcript sequences were specifically expressed in 33% of TNBC tumors and not in other breast cancer subtypes or control tissues. We report, for the first time, that the differentially expressed integrated EBV transcript sequences have the potential to generate novel miRNAs. Collectively, our findings contribute novel information towards the understanding of the differential expression, nature, and potential role of EBV gene transcript sequences present in various subtypes of breast tumors as compared to in control normal tissues.

We report here that total EBV-expressed RNA sequences were three-fold higher in breast tumors compared to in control breast tissues. The preferential expression of EBV-expressed sequences in breast tumors as compared to in control tissues is supported by some published observations. Lawson et al. reported a 5–6-fold higher EBV prevalence in breast cancer compared to in control normal breast tissues [85,86]. Similarly, Agolli et al. reported EBV prevalence in 27.9% of breast cancer cases as compared to in 8.02% of the control normal breast tissues [38]. Furthermore, EBV DNA was found to be prevalent in 36%, 23%, and 27% of breast cancer cases in Crotian, Jordanian, and Tunisian breast carcinoma cases, respectively; under these conditions, no control normal breast tissues were reported to be positive [87,88,89]. The prevalence of EBV DNA was also reported in 16.4% of Jordanian breast cancer cases as compared to in 3.3% of the controls [90]; additionally, 6.7% of Iranian high-grade breast cancer cases contained EBV DNA as compared to none of the controls [91]. Joshi et al. reported that breast cancer cases in Indian women were found to be 54.9% positive for EBNA expression by immunohistochemistry, while none of the control samples were positive; under these conditions, the mean antibody levels against EBNA1 were 2.90-fold higher in breast cancer cases as compared to the results for the controls [92]. Most of the studies on EBV in breast cancer, however, have delivered variable and contradictory results; this is due to the reliance of these studies on polymerase chain reaction (PCR) and in situ hybridization techniques [58,62,65]. The variable nature of the existing reports is also attributed to the existence of specific EBV strains in certain populations and geographical locations [25,70,71,72]. Nevertheless, our report on the three-fold higher EBV-expressed sequences in breast tumors as compared to the results for control breast tissue is supported by published reports on the higher levels and prevalence of EBV sequences in breast cancer [85]. Our ability to detect EBV total expressed sequences in all our experimental samples is in line with the fact that at least 90% of the population is latently infected with EBV, and breast tissue can express EBV genes [93]; it is also due to the use of the sensitive unbiased genome-wide RNA-seq approach [65].

RNA-seq and associated techniques represent the best approach for studies focused on understanding the role of EBV and similar viruses in breast cancer; this is due to the ability of this technique to determine the identity, structure, prevalence, and quantitation of genome-wide expressed sequences and transcripts [65]. The sensitivity of this technique enabled us to determine that the total EBV-expressed RNA sequences in our experimental system were associated with all tumor and control samples. This technique also enabled us to determine that the total EBV-expressed sequences were three-fold higher in breast tumors compared to in controls. Studies that have utilized the RNA-seq approach, however, have failed to detect significant expression of EBV transcripts in breast cancer cases. Fimereli et al. reported no significant viral EBV transcription detected in whole breast cancer transcriptomes, since only a small number of EBV viral sequences were detected [58]. This group also reported that no EBV DNA was detected in samples that were positive for EBV RNA. Khoury et al. and Fuentes-Pananá et al. both reported no EBV DNA or transcripts in breast tumors under conditions where EBV transcripts were found in gastric carcinoma tumors [59,60]. Finally, Tang et al. found EBV DNA in breast tumors, without active transcription [64]. All these observations suggest that the EBV sequence count in breast cancers is low compared to that in gastric carcinomas. These observations also reflect the natural variations in EBV sequences in the various heterogeneous breast cancer samples utilized, as well variations in method sensitivity [94]. The following existing reports support these statements: (1) No EBV complete genome has yet been found or sequenced in breast cancer cases, although this has been accomplished for other EBV-associated gastric and nasopharyngeal carcinomas [57,61,63,66,67,68,69]. This implies that there is no complete EBV genome in breast cancer. (2) A hit-and-run mechanism of oncogenesis has been proposed for EBV in breast cancer [40,73,74]; in this scenario, remnants of short EBV sequences remain integrated in the genome [75,76]. These short-integrated sequences will be hard to detect, either at the DNA level or as transcribed sequences; this may then account for at least some of the variable reporting that accompanies the detection by various methods, including short-read deep sequencing approaches. (3) The EBV sequences within the breast cancer tumors are variable due to tumor heterogeneity and to the fact that EBV is likely to display different stages of latency and integration, and is lost once the tumor has been initiated [34].

We report here the differential expression of 26 known EBV gene transcript sequences in breast cancer tumors as compared to in control normal breast tissues. These results were obtained by utilizing the HISAT2 aligner and the O tool for transcript assembly. Under our analysis conditions, EBV gene expression was detected from the EBV-positive C666 cell line, but no significant expression was detected from the EBV-negative HK1 cell line or from 28 known EBV genes in our experimental samples. The 26 differentially expressed gene transcript sequences localized to specific known EBV genes, as confirmed through Integrative Genomics Viewer (IGV) visualization. These sequences were displayed as short integrated transcript sequences varying from 11–21 nucleotides in length; the sequences could not be disregarded because they formed differentially expressed hotspots between those of the normal controls and breast tumors or breast cancer subtypes. This novel observation and occurrence is supported by other RNA-seq reports that found no significant expression of EBV full-length transcripts in breast cancer cases [58,59,60,64]. Furthermore, our findings on the expression of short integrated EBV transcript sequences as hotspots are similar to observations reported by Friedenson et al. [34,75,76]. Overall, our findings are consistent with the reported observation that the EBV genome and sequences can be lost from breast cancer cases during cancer progression, leaving only remnants of integrated EBV sequences [34,75,76] that may or may not generate EBV-expressed transcript sequences. Our novel findings demonstrate that some of these integrated EBV transcript sequences are expressed as functional hotspots that are differentially expressed between control normal breast tissues and breast cancer tumors and subtypes.

Four categories of 26 known EBV gene transcript sequences were differentially expressed in at least two breast cancer tumors as compared to in control normal breast tissues. In one category of these genes, two EBV gene (BOLF1, BPLF1) transcript sequences were expressed at higher levels in breast cancer tumors as compared to those for normal control breast tissues. The BOLF1 gene encodes a tegument protein that facilitates virus production during lytic infection, as well as during latency and the lytic cycle [95]. BPLF1, on the other hand, encodes a tegument protein that facilitates B-cell immortalization, immune evasion, and tumorigenesis, while disrupting the repair of DNA damage and causing genomic instability [96,97,98]. The expression of both genes is normally associated with the EBV lytic circle [95,98]. Neither gene has been previously implicated in breast tumor oncogenesis; however, BPLF1 has been found to be expressed in both immortalized B-cells and gastric cancer tumors [96]. Since these two genes are expressed in our breast cancer system as short-transcript sequences, the functional role of these transcript sequences remains to be determined.

In the next category of expressed genes, seven (BVRF1, BALF3, BFRF3, BYRF1, EBNA2, BKRF1, BKRF4) EBV gene transcript sequences were downregulated in breast cancer tumor subtypes compared to in normal breast tissue controls. Some expression of BVRF1 transcript gene sequences was detected in all samples. BVRF1 produces a minor capsid protein that interacts with BPLF1 to assist with binding tegument with capsid; no oncogenic effects have been reported [99].

BALF3 gene transcript sequences were expressed in 67% of the control tissues and 65% of tumors; the expression levels in tumors were predominantly downregulated compared to in the controls. BALF3 is an early lytic gene which encodes a terminase with nuclease activity and mediates mature virion production [100]. It is overexpressed in GC and NPC, where it has been shown to promote genomic copy number aberrations, cell migration, cell invasion, and spheroid formation [29]; BVRF1 produces a minor capsid protein that interacts with BPLF1 [28,57,100]. The role of this gene in breast cancer has not been determined [100]. BYRF1/EBNA-2 gene sequences were detected in 67% of control tissues and 76% and 65% of tumors, respectively; the expression levels were predominantly downregulated in tumors compared to in controls. BYRF1/EBNA-2 is most strongly expressed immediately following infection, after which it regulates expression of the viral gene LMP1 and plays a major role in the maintenance of viral latency [57,101,102]. It also plays a role in promoting genomic rearrangement, which leads to the tumorigenesis of lymphomas, although studies have shown it is not necessary for epithelial cell tumorigenesis [101]. A previous study of breast tumors found that the expression of EBNA-2 was exhibited in similar percentages of samples, but the expression level did correspond with positive estrogen receptor expression [103]. BKRF1 gene transcripts were expressed in 33% of control tissues compared to in 18% of tumors. The role and expression timing of BKRF1 have not been elucidated. BKRF4 gene transcript sequences were expressed in 33% of control tissues and in only 12% of tumors; otherwise, the expression levels were not significantly different in breast cancer tumors and control breast tissues containing the sequences. BKRF4 is a lytic early tegument protein gene that functions as a histone chaperone, downregulating DNA repair [29]. It was reported to be expressed in gastric carcinoma, but was not predicted to drive oncogenesis due to the low levels of expression [29,57]. BFRF3 is expressed in all control tissues and 94% of breast tumors, with significantly lower expression in breast tumors. BFRF3 is a lytic-activated capsid protein [104]. BFRF3 has been found to bind to the functional domain of p65, preventing activation of NF-кB via TNF-α and therefore reducing inflammatory cytokine response [105]. Entry into the lytic phase (dubbed the immediate early phase) is marked by the expression of BZLF1 and BRLF1, detected in our samples, which act as transcriptional activators [106,107]; however, several vital early genes necessary for the expression of late genes (BVLF1, BGLF4, BGLF3, and BcRF1) were not detected [108]. The molecular characterization of these breast tumors suggests that an abortive lytic cycle was involved, with “leaky” expression of late genes at low levels [28].

We detected tumor-specific expression of 17 EBV gene transcript sequences. Eight (BALF5, BZLF2, BALF4, EBNA3C, BALF2, EBNA3B, BFRF2, BRLF1) of these were specifically expressed in breast cancer tumors and not in control normal breast tissue; the other nine demonstrated breast cancer subtype-specific expression (and are discussed below). BALF5 gene transcript sequences were expressed in 35% of breast tumors. BALF5 is a lytic early gene previously found to be overexpressed in gastric carcinoma and nasopharyngeal carcinoma [57]. It functions as a viral DNase, playing a role in viral DNA synthesis, nucleocapsid maturation, and immune evasion, and also promoting oncogenesis [109]. BALF4, BRLF1, and EBNA-3B gene transcript sequences were all found in 41% of breast tumors, with no expression detected in controls. BALF4 is a leaky late lytic gene that encodes envelope glycoprotein B, which induces genomic aberration via centrosome activation; its expression is strongly correlated with cells lacking DNA replication genes in gastric cancer [28,57,109]. BALF4, along with BALF5, has been found to correlate with GC oncogenesis, specifically with expression of the JAK-STAT pathway [110]. BALF4 and EBNA-3B, along with BDLF4, are known to suppress apoptosis [111]. EBNA-3B, expressed in the latent phase, is considered a tumor suppressor gene. Cells with mutated or deleted EBNA-3B genes show greater rates of transformation and a reduced rate of detection by T-cytotoxic cells in B-cells [112]. Interestingly, EBNA3B, as well as BRLF1, were found to be expressed in gastric carcinoma [57]. BRLF1 is a lytic immediate early gene which serves as a transcription factor to initiate the EBV lytic replication program [29,109]. It is known to affect cell-cycle progression and the mitotic phase; it also induces chromosome mis-segregation [109]. Its expression correlated negatively with interferon expression [113]. EBNA-3C, BALF2, and BFRF2 gene sequences were expressed in 29% of breast tumors. EBNA3C is a latency gene that is present at low levels in GC tumors [57]. EBNA-3C hypermethylates are members of the Ras family, a collection of tumor suppressor genes [50]. BALF2 is an early lytic gene which produces a DNA-binding protein [114]. It interacts with Rab1 and directs the glycosylation of gp350/220 during capsid assembly [115]. BALF2 is overexpressed in gastric carcinoma, and elevated BALF2 expression correlates to an increased risk of NPC oncogenesis [57,116]. BFRF2 was the only one of the five early genes whose transcript sequences were found to be expressed in our analysis system; the expression of this gene was not reported among the lytic genes found to be expressed in various EBV associated cancers. It is an early gene which, along with BGLF4, BGLF3, BcRF1, and BVLF1, forms a viral pre-initiation complex required for the expression of EBV late genes [108]. BZLF2 gene sequences were found in 47% of breast tumors. BZLF2 is also a lytic EBV gene that encodes glycoprotein gp42, which is known to suppress MHC Class II complex expression in B-cells [28]. Its elevated expression in our tumor samples suggests that it may play a similar role in immune evasion within breast tumors. Many of the tumor-specific expressed genes, such as BZLF2 and EBNA-3B, promote oncogenesis by improving immune evasion; others, notably the BALF family genes, increase genomic instability. Other expressed tumor-specific genes (BRLF1 and BFRF2) are relevant for initiating viral replication, which has been shown to support tumorigenesis [117].

Nine (BSLF1, BSRF1, BARF0, BMRF1, BILF1, BCRF2, LF1, BGLF1, BZLF1) EBV gene transcript sequences were strongly upregulated in specific breast cancer tumor subtypes; no expression was observed in control breast tissues. EBV BMRF1 and BARF0 gene transcript sequences were preferentially expressed in 40% of HER2 breast cancer subtypes and not in triple-negative breast cancer subtypes. BARF0 gene transcript sequences were expressed in 33% of non-TNBC tumors, but were detected in 40% of HER2+ tumors at five-fold higher levels of expression. BARF0 is a latent protein that is expressed in breast cancer [56]. It has been found to predispose breast cancer cells to malignant transformation through activation of the HER2/HER3 signaling pathways [40,118]. BMRF1 is an EBV viral DNA polymerase processivity factor that promotes genetic instability [29]. BMRF1 and BARF0 were not highly expressed in other EBV-associated epithelial tumors [57], and detected sequences were not expressed as hotspots in our system; thus, these genes were not studied further. LF1 and BGLF1 gene sequences were further analyzed since they displayed differentially expressed hotspots. LF1 and BGLF1 EBV gene transcript sequences were expressed in 88% and 53% of the BC tumors, respectively, with no expression detected in normal control breast tissues; both gene transcript sequences were preferentially expressed in 80% of HER2+ BC tumors at approximately two-fold higher levels as compared to the average values of all tumors. BGLF1 is a tegument gene, and LF1 is a lytic early gene; both are expressed in gastric cancer [29,57]. No previous expression of LF1 and BGLF1 in breast cancer were reported, and the nature of its LF1 lytic function is also currently unknown [109]. Although BARF0 has previously been associated with the HER2+ BC subtype, no evidence of this association was found for LF1, BGLF1, and BMRF1. Thus, our finding on the association of these genes with the HER2+ BC subtype presents a novel concept, and it is in line with previous findings. The association between EBV infection and the HER2+ phenotype has been reported by other investigators. Lawson et al. reported that EBV infection stimulated the malignant transformation of breast epithelial cells through the activation of the HER2/HER3 signaling pathway [85]. Mekrazi et al. also reported that EBV DNA was more significantly associated with HER2+ tumors than with other subtypes of breast cancer [78]. Thus, our findings potentially contribute towards the mechanistic understanding of EBV oncogenesis involving the HER2 subtype of breast cancer.

Preferential expression or lack of expression of gene transcript sequences in some breast cancer subtypes and not others was observed for BILF1, BCRF2, and BZLF1. BILF1 and BCRF2 were strongly expressed in non-TNBC breast cancer tumors, with no detectable expression in TNBCs and with low or undetected expression in HER2 tumors. BZLF1 gene transcript sequences were expressed in 33% of TNBC and non-TNBC tumors and were not detected in HER2 tumors. BILF1 is a lytic early gene that was overexpressed 75-fold in gastric carcinoma [57]; it modulates immune evasion by degrading the MHC Class I structure [119]. BCRF2 is a lytic protein that interacts with and stabilizes BSRF1 to facilitate virus envelopment, thus increasing virus infectivity and the production of virions [120]. BZLF1 is an immediate early gene that is a principal activator of EBV lytic gene expression [28,29]. It controls the shift of EBV from the latent phase to the lytic phase, thus controlling the lytic genes encoding the core replication machinery [121]; these lytic genes include BALF5, BALF2, BSLF1, and BMRF1 found to be upregulated in our experimental system [109]. BZLF1 is expressed in Burkitt lymphoma, nasopharyngeal carcinoma (NPC), and gastric carcinoma [28,57]; it has also been reported to be expressed in breast cancer, in line with our results [56]. The tumor-specific expression of BILF1 and BZLF1, which act on the MHC I and II pathways, demonstrates the potential mechanisms for significantly improved immune evasion by EBV-infected tumor cells.

BC subtype-specific expression of BSLF1 and BSRF1 EBV transcript sequences was observed primarily in the TNBC BC subtypes and the TNBC and HER2 subtypes, respectively. BSRF1 (75,254–75,265) and BSRF1 (74,938–74,958) gene transcript sequences were uniquely expressed in 20% of HER2+ tumors and in 33% of the triple-negative BC tumors, respectively; this demonstrates the expression of different specific BSRF1 gene transcript sequences in the TNBC subtypes as compared to in the HER2 breast cancer tumor subtype. The BSRF1 gene encodes a tegument protein that plays a role in viral maturation and release; it forms a complex with BBRF2 that facilitates viral envelopment. It also modulates the host immune response [29,120,122], but no direct oncogenic effects in breast cancer have been identified. BSLF1 gene transcript sequences were also specifically expressed in 33% of TNBC tumors, but these expressed sequences were not detected in other breast cancer subtypes or control tissues. BSLF1 is a viral primase which forms part of the helicase–primase complex that comprises the core replication machinery of BALF5, BALF2, BBLF2/3, BBLF4, BMLF1, BSLF1 and BMRF1 [28,109]. BSLF1 is expressed in gastric carcinoma [109]. Since the TNBC subtypes are heterogeneous and exist in six different molecular subtypes, these results point to possible TNBC subtype-specific expression of the BSLF1 gene transcript sequences; this concept remains to be investigated.

The differentially expressed EBV gene transcript sequences were found to be composed of short EBV integrated sequences, and functional analysis revealed that these sequences exhibited the potential to create novel EBV miRNAs. Our observations on the integration of EBV short sequences is supported by other reports. The EBV genome likely disappears from the tumor cells after malignant transformation, since its presence is no longer required; what remains then are short remnant sequences that are integrated into the host genome and its transcripts [34,40,85,123,124]. Furthermore, La Frazia et al. reported that the EBV integration breakpoints within tumors were distributed throughout the EBV genome, particularly at terminal repeats. The integration sites involved CpG islands, leading to genomic instability, thus playing a role in tumor progression [73]. This report is in line with our observations on the EBV gene transcript sequences, which are GC rich. Our findings that the differentially expressed EBV gene transcript sequences have the potential to generate novel miRNAs is an important and novel concept. Mundo et al. proposed that the detection of microRNAs is the most specific and sensitive tool to recognize the presence of the disappearing EBV sequences in tumors [125]. Our unpublished observations on miRNA profiling in breast cancer tumors utilizing RNA-seq supports this concept; our studies have readily revealed the presence of novel differentially expressed miRNAs that map to the EBV genome and contain fused human sequences. This finding is also supported by the recent report on the association of breast cancer-integrated EBV genome fragments with piRNA genes [75]. Thus, our observations on the capacity for the integrated differentially expressed short EBV transcript sequences to generate novel miRNAs provides a mechanism to explain the continued EBV influence on tumorigenesis in the face of EBV sequences disappearing from the tumors.

## 5. Conclusions

In summary, we report, for the first time, the differential expression of 26 EBV gene transcript sequences in breast cancer tumors and among specific breast cancer subtypes as compared to in control breast tissues. Since short integrated EBV gene transcript sequences are hard to detect, our findings provide an explanation for why other investigators have previously failed to detect them. We also report, for the first time, that the differentially expressed integrated EBV transcript sequences have the potential to generate novel miRNAs. MicroRNAs are important because they mediate biological processes in EBV oncogenesis, from initiation to metastasis across genetic and epigenetic mechanisms [126]. The role and mechanism of EBV oncogenicity in breast cancer remains unknown [127]. The EBV genome and sequences have been reported to disappear from breast cancer tumors, leaving only short integrated EBV sequences that may or may not be expressed at the RNA level [34]. Our findings provide a potential explanation regarding how EBV can continue to exert its oncogenic effect on breast cancer tumors and its subtypes after most of the EBV genome disappears. Thus, collectively, our findings contribute novel information towards the understanding of EBV’s role in breast cancer etiology and breast cancer subtype specific heterogeneity, as well as its pathogenic mechanisms. A study of additional datasets to increase statistical power is planned. Our future studies will examine the identity of the integration sites for the EBV transcript-expressed sequences, as well as the mechanisms by which novel EBV miRNAs originate from these sites. Additional future studies will focus on the functional role of these novel EBV miRNAs in the EBV oncogenic process in breast cancer.

## Data Availability

Publicly available short-read data (PRJNA487983, PRJNA227137, and PRJNA482141) was used for analysis.

## Supplementary Materials

The following supporting information can be downloaded at: https://www.mdpi.com/article/10.3390/genes16070756/s1, Figure S1: Expression of all EBV gene transcript sequences in normal controls and in various breast cancer tumor subtypes; Figure S2: EBV Gene transcript sequences downregulated in breast tumors; Figure S3: EBV Gene transcript sequences expressed in breast tumors and not in normal breast control tissues; Figure S4: EBV Gene transcript sequences with unique or upregulated expression in specific breast tumor subtypes; Figure S5: Method for EBV miRNA functional analysis.
