# Supplementary material for: Differential Expression of Epstein–Barr Virus Sequences in Various Breast Cancer Subtypes
Source: Genes (Basel). 2025 Jun 27;16(7):756. doi: 10.3390/genes16070756 (PMC12294798; doi:10.3390/genes16070756)
Supplement: Supplementary file 1 [file genes-16-00756-s001.zip › Manuscript ID_Genes 3694619 supplemental figures _and legends 062725.pdf]

MANUSCRIPT ID: GENES 3694619

SUPPLEMENTAL FIGURES

Supplemental Figures

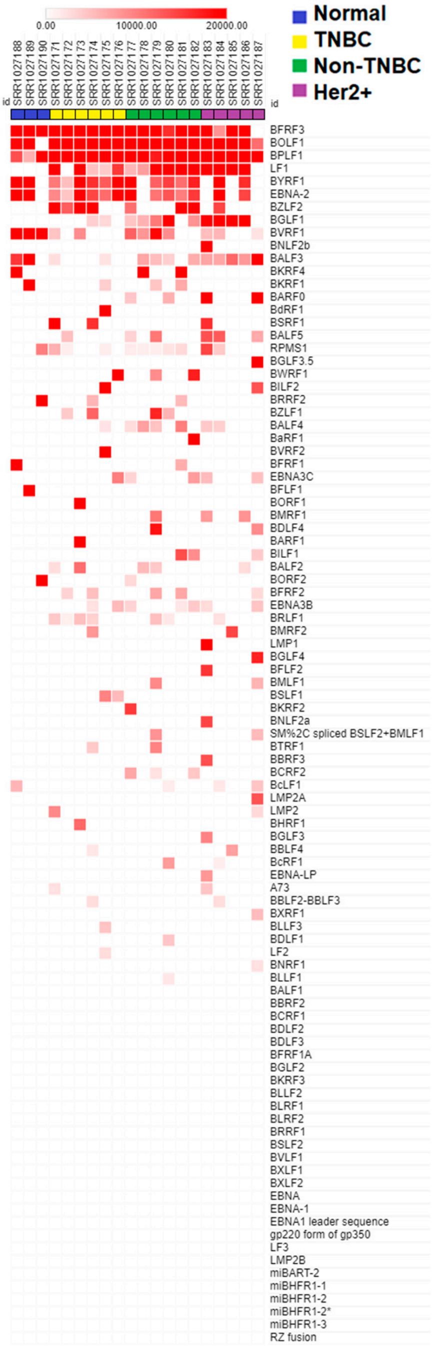

Supplemental Figure S1: Expression of all EBV gene transcript sequences in normal controls and in various breast cancer tumor subtypes. Trimmed raw RNA-seq reads from normal and various breast cancer subtypes (as shown in figure 1B) were aligned against the EBV genome in FASTA format using HISAT2. Bam file reads were assembled into transcripts and quantified by utilizing the StringTie tool and the EBV genome in gene feature format. A heat map depicting the relative quantity (in FPKM values) of all EBV gene transcript expressed sequences is shown, ordered from greatest to least mean level of expression.

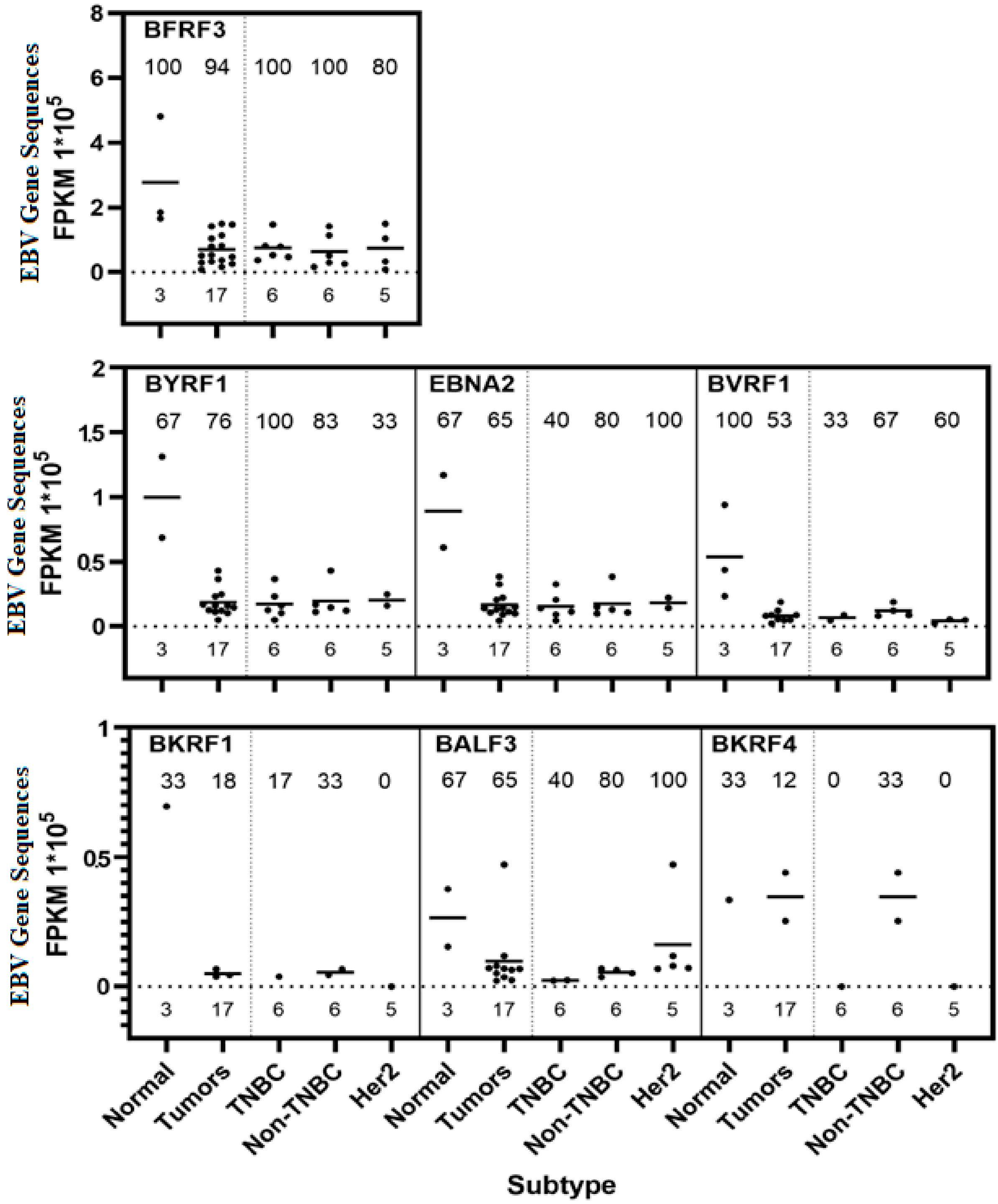

Supplemental Figure S2: EBV Gene transcript sequences downregulated in breast tumors. EBV gene transcript sequences quantitated (as in figure 2D) for each sample are shown in graphs and grouped by similar levels of FPKM expression. The number of the samples analyzed for the graphical representation for each subtype is shown above the x-axis. The % of the positive samples expressing each EBV gene sequence for each subtype is shown at the top of each the graph.

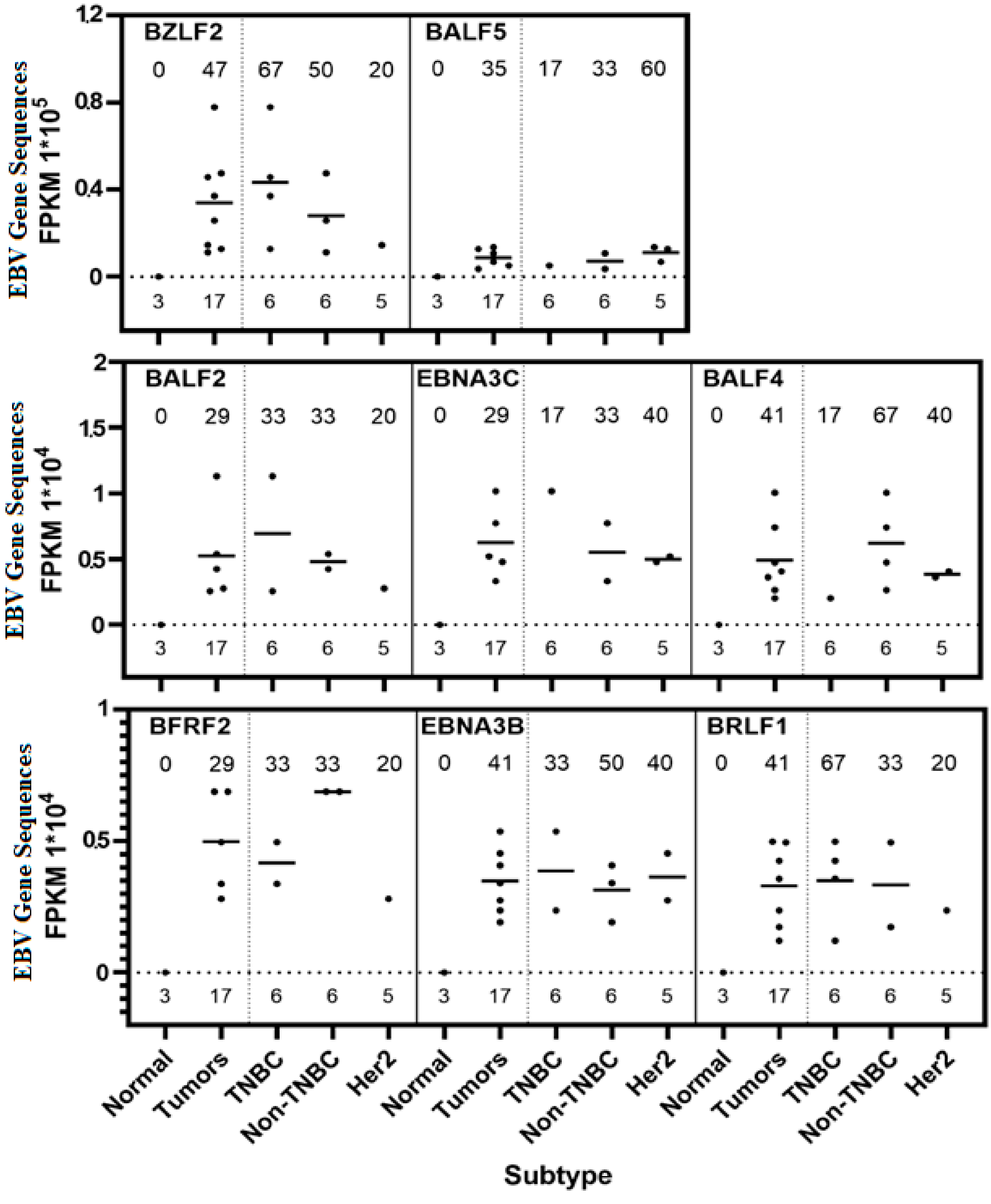

Supplemental Figure S3: EBV Gene transcript sequences expressed in breast tumors and not in normal breast control tissues. EBV gene transcript sequences quantitated (as in figure 2B) for each sample are shown in graphs and grouped by similar levels of expression in FPKM. The number of the samples analyzed for the graphical representation for each subtype is shown above the x-axis. The % of the positive samples expressing each EBV gene sequence for each subtype is shown at the top of each the graph.

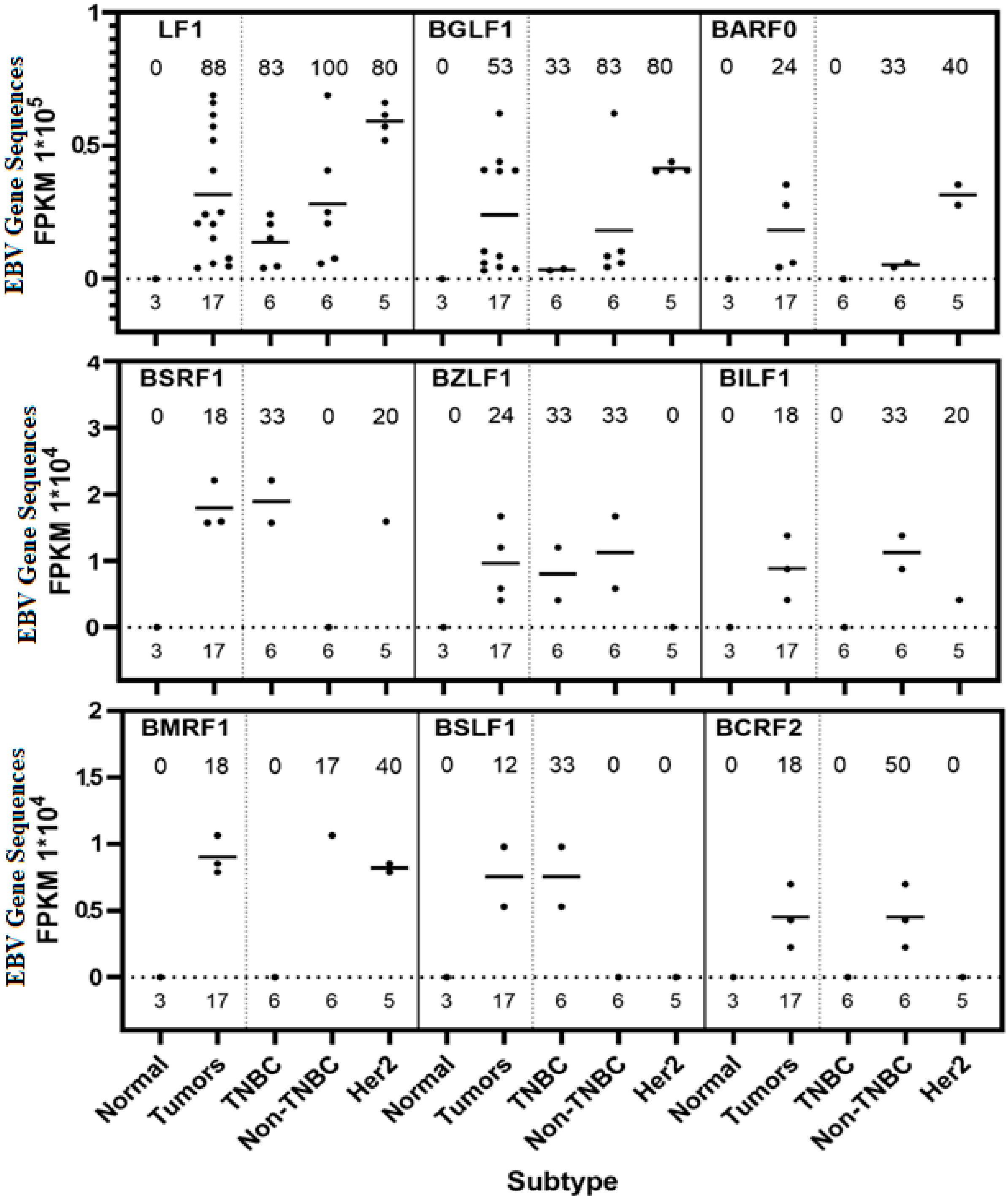

Supplemental Figure S4: EBV Gene transcript sequences with unique or upregulated expression in specific breast tumor subtypes. EBV gene transcript sequences quantitated (as in figure 2C) for each sample are shown in graphs and grouped by similar levels of expression in FPKM. The number of the samples analyzed for the graphical representation for each subtype is shown above the x-axis. The % of the positive samples expressing each EBV gene sequence for each subtype is shown at the top of each the graph.

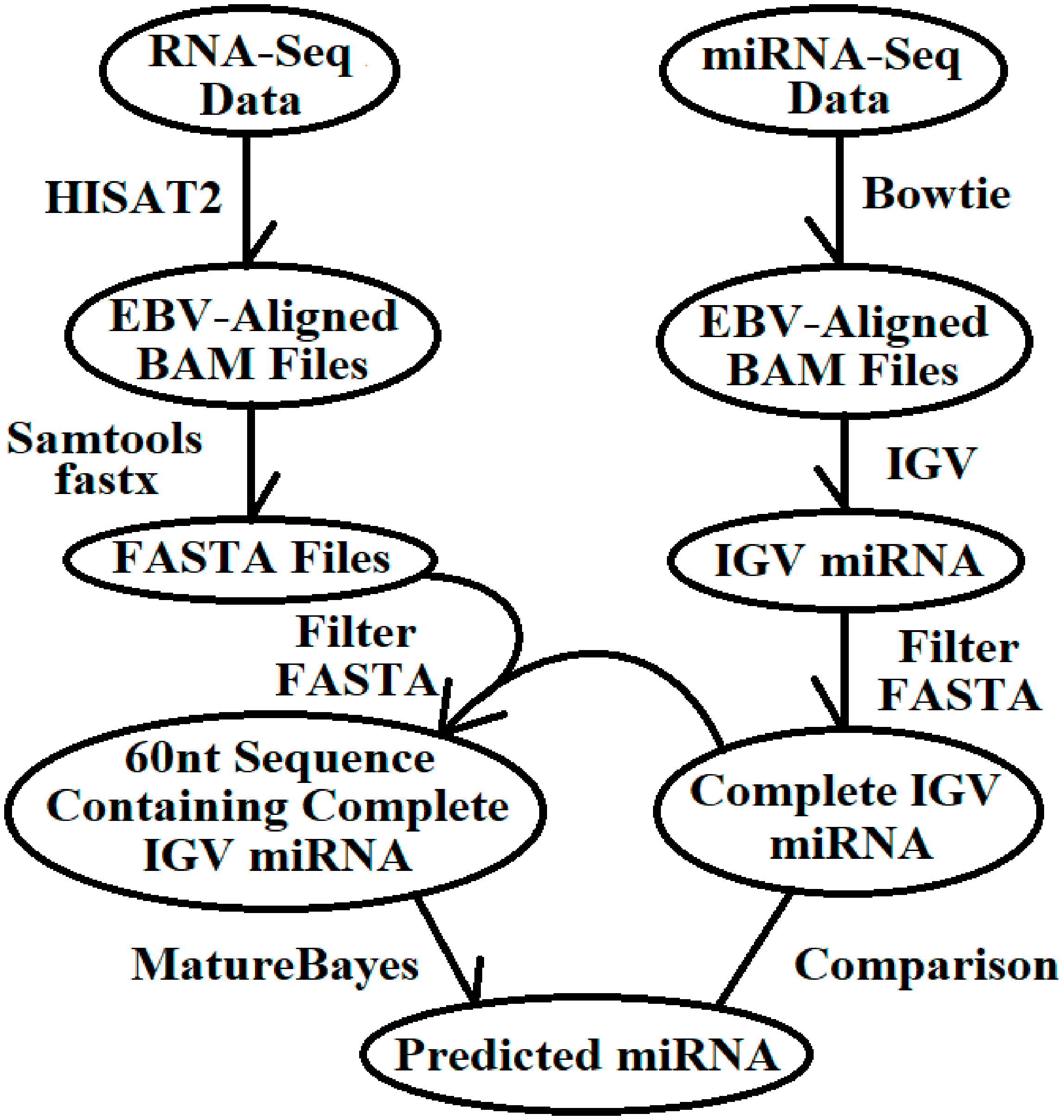

Supplemental Figure S5: Method for EBV miRNA functional analysis. Flowchart representation of method used for functional analysis of EBV miRNAs produced by EBV sequence hotspots (Methods section 2.4). miRNA-seq data was aligned against the EBV genome. Actual sequence of select EBV hotspot miRNAs was recorded using IGV. Complete sequence of IGV miRNA was taken from EBV-aligned miRNA BAM files. 60nt sequence containing the complete miRNA sequence was taken from RNA-seq FASTA sequence converted from BAM file. MatureBayes was used to predict mature miRNA to confirm production of miRNA by RNA-seq sequences.
